# ACCESS TO CARE ROUNDS: A UNIQUE FORUM FOSTERING HEALTH SYSTEM AND SOCIAL SERVICE PARTNERSHIPS FOR OLDER VETERANS

**DOI:** 10.1093/geroni/igz038.1839

**Published:** 2019-11-08

**Authors:** Kathryn A Nearing, Hillary D Lum, Heather Kamper, Kaily A Cannizzaro, Courtney Bauers, Joleen C Sussman

**Affiliations:** 1 University of Colorado Anschutz Medical Campus, Division of Geriatrics, Aurora, Colorado, United States; 2 Area Agency on Aging, Denver Regional Council of Governments, Denver, Colorado, United States; 3 Rocky Mountain Mental Illness Research Education Clinical Center for Suicide Prevention, Aurora, Colorado, United States; 4 Denver-Seattle Center of Innovation for Veteran-Centered and Value-Driven Care, Aurora, Colorado, United States; 5 Rocky Mountain Regional VA Medical Center, Aurora, Colorado, United States

## Abstract

Colorado Veteran Community Partnership (VCP) aims to connect Rocky Mountain Regional VA Medical Center front-line teams with diverse community partners to create integrated networks of support for older Veterans with complex needs and their family members and caregivers. To accomplish this goal, VCP launched Access to Care Rounds in January 2018 to build bridges between the healthcare system and community-based organizations. Each Access to Care Rounds features a cross-sector panel that discusses specific efforts to link a medically-complex, older Veteran to resources. This model was developed with stakeholder input and has highlighted topics related to chronic pain management, suicide prevention, homelessness, adult protective services, transportation, home-based primary care, hospice care, and firearm safety. Each Access to Care Rounds focuses on connecting VCP members, sharing expertise and resources, and highlights lessons learned related to care coordination, communication, and key processes that others can adopt/adapt to better serve older Veterans. On average, 30 individuals attend each session. Access to Care Rounds draw diverse audiences representing social services, mental health and other healthcare specialties. The latter include Social Workers (47%), Physicians (11%), Psychologists (8%), Registered Nurses (6%), and students/trainees (6%). Participants receive a description of the Veteran situation; the names, credentials, organizational affiliations and roles/expertise of each panelist; and, a resource list relevant to the constellation of issues addressed to enhance access to information and resources. Over 38% of respondents to session evaluations reported intentions to change their professional practice as a result of what they learned during an Access to Care Rounds.

